# The effect of family sports environment on physical exercise behavior of junior high school students—the chain mediating effect of perceived family support and self-efficacy

**DOI:** 10.3389/fpsyg.2026.1821116

**Published:** 2026-07-07

**Authors:** Tingxiao Zhang, Wenlan Zhou, Qi Wu, Wanyang Ren, Yuxiang Fang

**Affiliations:** School of Physical Education, Liaoning Normal University, Dalian, China

**Keywords:** family sports environment, junior high school students, perceived family support, physical exercise behavior, self-efficacy, the chain mediating effect

## Abstract

**Aim:**

This investigation seeks to elucidate the mechanistic pathways through which perceived family support and self-efficacy mediate the relationship between the family sports environment and physical exercise behavior among junior high school students. Ultimately, this research aims to establish a robust theoretical foundation and offer practical insights to foster the cultivation of sustained physical activity habits, thereby enhancing the overall physical health and wellbeing of this demographic.

**Methods:**

A questionnaire survey was conducted on 729 junior high school students using validated scales, and structural equation modeling (SEM) was adopted to test the hypothesized chain mediation model, with Harman's single-factor test used to control common method bias.

**Results:**

(1) The family sports environment, perceived family support, and self-efficacy of junior high school students were all at a relatively low level, and their physical exercise behavior was generally poor, with significant demographic differences in all variables (gender, family structure, etc.). (2) There were significant positive correlations among the family sports environment, perceived family support, self-efficacy, and physical exercise behavior, and all three variables had a significant positive predictive effect on physical exercise behavior, with the family sports environment exerting the strongest effect. (3) For the overall sample, perceived family support and self-efficacy did not play a mediating or chain mediating role between the family sports environment and physical exercise behavior; the chain mediating path of “family sports environment → perceived family support → self-efficacy → physical exercise behavior” was only significant in nuclear families.

**Conclusion:**

This study reveals the internal mechanism and boundary conditions of the family sports environment influencing junior high school students' physical exercise behavior, and provides theoretical references and practical suggestions for families, schools and communities to jointly construct a sports support network and improve adolescents' physical exercise behavior.

## 1 Introduction

Physical exercise is a core carrier for promoting the physical and mental health of adolescents, and the cultivation of sustained physical exercise behavior in junior high school students—who are in a critical period of physical and mental development—bears important significance for their lifelong health and the improvement of the overall quality of the national human resources. However, the current physical exercise status of junior high school students is not optimistic: physical fitness indicators such as endurance and strength improve slowly, overweight and obesity problems are prominent, exercise frequency is low, exercise motivation is insufficient, and there is an over-reliance on physical education classes at school. These problems not only restrict the immediate physical health development of junior high school students but also hinder the formation of their lifelong exercise habits, becoming a practical dilemma that needs to be solved in adolescent health promotion.

The family is the primary living and educational environment for adolescents, and the family sports environment, as a key external environmental factor affecting adolescent physical exercise behavior, refers to the comprehensive behavioral, physical, and psychological conditions within the family that influence members‘ participation in physical exercise. Existing sports psychology and social cognitive theory research have confirmed that the external environment is an important predictor of individual behavior, and the family sports environment can directly shape adolescents' sports participation willingness and behavior through parental behavioral demonstration, sports facility provision, and positive psychological atmosphere construction. In addition to the direct effect, the influence of the family sports environment on physical exercise behavior is often transmitted through internal psychological variables: perceived family support, as the subjective experience of emotional, material, and behavioral support from family members, acts as a psychological bridge connecting the external family environment and individual exercise behavior; self-efficacy, defined as an individual's subjective judgment of their ability to complete physical exercise tasks, is a core internal driving force for initiating and maintaining physical exercise behavior according to Bandura's social cognitive theory.

Perceived family support is the emotional, material, and behavioral support that junior high school students subjectively feel from family members. Self-efficacy is an individual's subjective judgment of their ability to complete physical exercise tasks. As important psychological variables, the two play a key mediating role between the external environment and individual behavior. Existing research shows that a positive family sports environment can significantly promote adolescents‘ sports participation, and perceived family support and self-efficacy also have positive effects on physical exercise behavior, respectively. However, the internal mechanism of action among the three, especially the chain mediating relationship, has not been fully revealed. Therefore, deeply exploring the influencing path of the family sports environment on junior high school students' physical exercise behavior and analyzing the chain mediating effect of perceived family support and self-efficacy have important theoretical and practical value for solving the physical exercise dilemma of junior high school students, formulating targeted intervention strategies, and improving the physical health level of adolescents.

## 2 The chain mediating hypothetical model of family sports environment and junior high school students' physical exercise behavior

### 2.1 Predictive effects of family sports environment on junior high school students' physical exercise behavior

The family sports environment will directly affect students‘ physical exercise behavior. When the family sports environment is excellent, it can enhance students' enthusiasm for participating in physical exercise, and then promote them to develop good physical exercise behaviors (Timperio et al., 2013). The family sports environment can significantly promote students‘ physical exercise behavior, especially in the urban student group (Ge, 2024). In the family sports behavior environment of junior high school students, the more encouragement parents give to their children, and the higher the exercise support and logistics support, the more positive the attitude of junior high school students toward physical exercise, and their physical exercise behavior will also be better (Wu, 2024). The family sports environment plays an irreplaceable and important role in promoting the physical health of teenagers and cultivating their sports behavior. However, there are generally problems in the construction of the family sports environment, such as the imbalance between public sports services and family sports configuration supply, the impact of family situation differences on teenagers' physical exercise behavior, the differences in educational concepts caused by parents‘ educational levels, and the imperfect collaborative mechanism of multiple subjects, which in turn affect the effective formation and sustainable development of teenagers' physical exercise behavior (Fu et al., 2025). Based on this, we propose Hypothesis H1: The family sports environment has a positive and significant predictive effect on the physical exercise behavior of junior high school students.

### 2.2 Prediction of perceived family support and physical exercise behavior

Perceived family support, as an individual's subjective experience of family emotional and resource support, is widely regarded as an important psychological bridge connecting the family environment and individual behavior. Parents‘ educational level, positive attitude toward sports, and behavior of participating in sports activities will all affect students' physical exercise behavior (Riso et al., 2019; Rhodes et al., 2018; Ahmed et al., 2016). The sports facilities and opportunities provided by the family will also directly affect students‘ physical exercise behavior (Zhang and Hasibagen, 2022). At the same time, the social status and economic conditions of the family will also affect students' physical exercise behavior (Pan et al., 2022). In addition, family support has a positive and significant impact on the physical exercise behavior of junior high school students. Its role in the process of changing the exercise behavior of junior high school students can further promote the development of junior high school students‘ physical exercise activities and improve their physical health (Zheng, 2021). At the same time, good family support can effectively improve students' physical exercise behavior (Zhao, 2023; Pang, 2024). Based on this, we propose Hypothesis H2: Perceived family support has a positive and significant predictive effect on physical exercise behavior.

### 2.3 Prediction of self-efficacy and physical exercise behavior

Self-efficacy has been proven to be a key psychological variable in predicting and explaining behavior in the field of physical exercise. Overall, there is a significant positive correlation between self-efficacy and physical exercise behavior. Among them, self-efficacy can effectively predict the frequency, intensity, and duration of college students' physical exercise (Xu, 2023). Existing research shows that college students' exercise self-efficacy can not only directly and positively affect physical exercise behavior, but also indirectly affect exercise behavior through the chain mediating effects of “physical learning investment” and “exercise commitment”, indicating that self-efficacy promotes the maintenance of exercise behavior step by step by stimulating learning motivation and enhancing behavior commitment (Ye, 2024). Research has confirmed that there is a positive correlation between college students' self-efficacy and physical exercise behavior (Li, 2024). At the same time, female college students' physical exercise behavior is positively correlated with their general self-efficacy level, and those with high self-efficacy are more inclined to actively participate in physical exercise (Zhang, 2025). These studies together show that enhancing adolescents' self-efficacy is an effective psychological intervention approach to promoting their physical exercise behavior. On this basis, we propose Hypothesis H3: Self-efficacy has a positive and significant predictive effect on physical exercise behavior.

### 2.4 Prediction of the chain mediating effect of the family sports environment, perceived family support, and self-efficacy on junior high school students' physical exercise behavior

**Figure 1:**
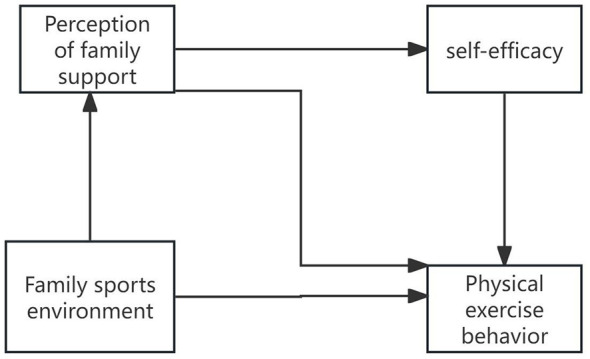
Diagram of the hypothetical model.

In the study of the relationship among the family sports environment, self-efficacy, and physical exercise behavior, it was found that the family sports environment can not only directly and positively predict the physical exercise behavior of junior high school students but also have an indirect impact through the mediating role of self-efficacy. This indicates that a good family sports environment promotes adolescents‘ active participation in physical exercise by enhancing their confidence in sports and the belief in overcoming exercise barriers, verifying the “psychological bridge” role of self-efficacy between family support and exercise behavior (Zhang, 2024). In addition, in the study of the relationship among the family sports environment, perceived family support, and physical exercise behavior, it was found that the family sports psychological environment can not only significantly and positively predict parents' support behavior for their children's sports activities but also directly promote the physical exercise behavior of junior high school students. More importantly, the family sports psychological environment can also indirectly affect the physical exercise behavior of junior high school students through the mediating effect of parental support. This shows that a positive psychological atmosphere within the family can be transformed into specific support behaviors by parents, and after being perceived by their children, these behaviors ultimately prompt them to participate in physical exercise more actively. On this basis, we propose Hypothesis H4: Perceived family support and self-efficacy can jointly play a chain mediating role between the family sports environment and the physical exercise behavior of junior high school students. The hypothetical model is shown in Figure 1.

## 3 Methodology

### 3.1 Research subjects

The research subjects are the effects of family sports environment on physical exercise behavior of junior high school students, and the chain mediating effect of perceived family support and self-efficacy is deeply examined. A total of 729 junior high school students were selected by means of stratified cluster random sampling.

### 3.2 Research method

This study adopted a cross-sectional survey design to explore the relationship between family sports environment, perceived family support, self-efficacy and physical exercise behavior of junior high school students, and to test the chain mediating effect of perceived family support and self-efficacy, as well as the heterogeneous characteristics of the mediating effect in different family structure groups. Harman's single-factor test was used to control the common method bias, and reliability and validity test, correlation analysis and structural equation modeling (SEM) were used for statistical analysis. The interview method was used to supplement the construction of the research framework and the selection of measurement tools, and the results of the interview were used for qualitative analysis to provide a theoretical and practical basis for the design of the questionnaire survey and the interpretation of the empirical results.

#### 3.2.1 Literature review method

Using keywords such as “family sports environment”, “perceived family support”, “self-efficacy”, “physical exercise behavior”, and “junior high school students”, a search was conducted in the database. Understand the conceptual definitions of “family sports environment”, “perceived family support”, “self-efficacy”, and “physical exercise behavior” at home and abroad, and sort out the research status in this field to lay a solid theoretical foundation for this study.

#### 3.2.2 Interview method

This study used the structured interview method to interview experts and professors at relevant universities, and sought their suggestions on the conceptual definitions, measurement tool selection, and data analysis of “family sports environment”, “perceived family support”, “self-efficacy”, and “physical exercise behavior”, providing first-hand information for this study. At the same time, this study used the semi-structured interview method. The interviewees were selected by purposive random sampling. Students and parents from 3 families in each of the first to third grades of 6 different schools were interviewed. An interview outline needed to be prepared in advance, and communication was carried out with physical education teachers in each grade to clarify the plan and purpose of the interview, and to understand the family sports environment, parents‘ support for physical exercise, and the actual situation of students' participation in physical exercise. Then, the information obtained from the interviews was coded and sorted for analysis.

#### 3.2.3 Questionnaire survey method

This study used the “Questionnaire on Junior High School Students' Family Sports Environment” revised by Zheng Yanan, the “Perceived Social Support Scale (PSSS)” adapted by researchers such as Huang Li, the “General Self-Efficacy Scale (GSES)” revised by Wang Caikang, and the “Physical Activity Rating Scale (PARS-3)” compiled by Liang Deqing et al. These scales have passed multiple tests and have good reliability and validity. In order to deeply understand the situation of junior high school students, in addition to distributing mature scales, information such as parents' education level, income, junior high school students' gender, grade, whether they are only children, and health status was added.

#### 3.2.4 Mathematical statistics method

The data of the recovered valid questionnaires were entered into Excel to establish an original database, and then imported into the SPSS 26.0 statistical software for analysis. First, the reliability and validity tests of the measurement tools were carried out. The Cronbach's α coefficient was used to evaluate the internal consistency reliability of the scales, and the confirmatory factor analysis was used to test the construct validity of each latent variable to ensure the fitness and reliability of the measurement model. Secondly, basic statistical analysis was carried out, including descriptive statistics of each core variable, examining the differences in demographic variables such as gender and grade of the sample, and using Pearson correlation analysis to test the pairwise correlation relationships among the family sports environment, perceived family support, self-efficacy, and physical exercise behavior, providing a preliminary basis for the subsequent model construction. Finally, based on the proposed chain mediation model in the study, the structural equation model (SEM) method was used to test the chain mediation path of “family sports environment—perceived family support—self-efficacy—physical exercise behavior”. Using the Bootstrap sampling method recommended by Professor Wen Zhonglin, the significance of each indirect effect path was tested under a 95% confidence interval to scientifically verify the chain mediating role of perceived family support and self-efficacy in the relationship between the family sports environment and middle school students' physical activity behavior.

Mediation effects were tested using structural equation modeling in AMOS 24.0. The chain mediation model was examined following the bootstrap method. An indirect effect was considered significant if the 95% CI did not include 0. Multiple-group SEM was used to test the moderating role of family structure.

### 3.3 Research instruments

#### 3.3.1 Family sports environment scale

In the research process, the parent questionnaire revised by Zheng Yanan was adopted, which was based on the “Junior High School Students' Family Sports Environment Questionnaire” compiled by Yang Jiapeng in 2017. This questionnaire consists of 74 questions and is composed of three dimensions: family sports behavior environment, family sports physical environment, and family sports psychological environment. The questionnaire uses a Likert 5-point scoring method, and the higher the score, the better the situation of the family sports environment.

#### 3.3.2 Perceived family support scale

The “Perceived Social Support Scale (PSSS)” adapted by researchers such as Huang Li was adopted. This questionnaire consists of two parts: endogenous support and exogenous support. In this survey, the family support part was selected, which has a total of 4 questions. The scale uses a Likert 7-point scoring method, and the higher the score, the higher the degree of perceived family support.

#### 3.3.3 Self-efficacy scale

The “General Self-Efficacy Scale (GSES)” revised by Wang Caikang was adopted. This scale is a single-dimensional scale with a total of 10 items. The scale uses a Likert 4-point scoring method, and the higher the score, the stronger the self-efficacy.

#### 3.3.4 Physical exercise behavior scale

The “Physical Activity Rating Scale (PARS-3)” revised and compiled by scholars such as Liang Deqing was adopted. This scale is composed of three dimensions: exercise time, exercise frequency, and exercise intensity. In the scale, the three dimensions of exercise time, exercise frequency, and exercise intensity are divided into five levels according to size, and the higher the score, the better the physical exercise behavior.

### 3.4 Test of reliability and validity

Due to research needs and different subject groups, it is necessary to conduct reliability and validity tests on the mature scales involved in this study again. Moreover, since the “Physical Exercise Behavior Scale” has only 3 questions, which does not meet the variable number requirements for factor analysis, only the “Family Sports Environment Scale”, “Perceived Family Support Scale”, and “Self-Efficacy Scale” were subjected to questionnaire tests. The questionnaires were randomly distributed to 30 students in each of the first, second, and third grades of junior high school, and the questionnaires were collected on the spot after testing. Cronbach's α and confirmatory factor analysis (CFA) were used to test the reliability and validity of the three scales. The results are shown in Table 1. It was found that the Cronbach's α of the three scales was better than 0.8, and all indicators of validity were within a reasonable range, indicating that the reliability and validity of the scales were relatively high and suitable for use in this study.

**Table 1:** Test of reliability and validity.

Categories	Reliability	Validity					
	Cronbach's α	χ^2^/df	*p*	GFI	RMSEA	RMR	CFI
Family sports environment scale	0.979	1.901	0.000	0.917	0.091	0.029	0.917
Family sports environment scale	0.829	1.278	0.049	0.986	0.056	0.049	0.995
Self-efficacy scale	0.931	1.477	0.034	0.904	0.073	0.038	0.973

## 4 Research results

### 4.1 Control and test of common method bias

To examine whether common method bias existed in this study, the most commonly used Harman's single-factor test was adopted. The results (shown in Table 2) indicated that the first factor accounted for 36.447% of the variance, which was below the 40% critical threshold. This suggested that no severe common method bias was present in the present study.

**Table 2:** Variance explained rates table.

Factor	Unrotated eigenvalue			Unrotated variance (%)			Unrotated cumulative (%)		
	Unrotated eigenvalue	Unrotated variance (%)	Unrotated cumulative (%)	Rotated eigenvalue	Rotated variance (%)	Rotated cumulative (%)	Factor no.	Unrotated eigenvalue	Unrotated variance (%)
1	37.176	36.447	36.447	37.176	36.447	36.447	13.673	13.405	13.405
2	1.966	1.928	38.375	1.966	1.928	38.375	12.603	12.356	25.761
3	1.841	1.805	40.181	1.841	1.805	40.181	11.718	11.488	37.249
4	1.797	1.761	41.942	1.797	1.761	41.942	3.867	3.792	41.040
5	1.746	1.712	43.654	1.746	1.712	43.654	2.472	2.423	43.464
6	1.691	1.658	45.311	1.691	1.658	45.311	1.877	1.840	45.304

### 4.2 Descriptive analysis of family sports environment, perceived family support, self-efficacy and physical exercise behavior of junior high school students

#### 4.2.1 Descriptive analysis of family sports environment

As shown in Table 3, the mean score of family sports behavioral environment was 129.427, lower than the median of 145. For family sports physical environment, the mean was 92.095 and the median was 103. Meanwhile, family sports psychological environment had the lowest mean score of 54.018, with an SD of 13.004 and a median of 60. In terms of total scores, the mean score of family sports environment was 275.539, slightly lower than the median of 310, indicating an acceptable overall level but a left-skewed distribution. This implied that the family sports environment of some students was relatively poor, pulling down the mean. The SD of 60.642 revealed large individual differences in the family sports environment among junior high school students.

**Table 3:** Descriptive statistics of family sports environment among junior high school students (*n* = 729).

Variable	Min	Max	Mean	SD	Median
Family sports behavioral environment	70.000	162.000	129.427	28.078	145.000
Family sports physical environment	54.000	120.000	92.095	20.731	103.000
Family sports psychological environment	23.000	73.000	54.018	13.004	60.000
Family sports environment (total)	168.000	342.000	275.539	60.642	310.000

#### 4.2.2 Descriptive analysis of perceived family support

It can be seen from the data shown in Table 4 that the average value of perceived family support is 18.499, which is slightly lower than the median of 21.000. At the same time, the standard deviation of junior high school students' perceived family support is 6.027. Combined with the range from the minimum value of 5 to the maximum value of 27, it reflects that there are huge differences in the perception of family support among different junior high school students. Some students can fully perceive family support, while others cannot.

**Table 4:** Descriptive statistics of perceived family support among junior secondary school students (*n* = 729).

Variable	Min	Max	Mean	SD	Median
Perceived family support	5.000	27.000	18.499	6.027	21.000

#### 4.2.3 Descriptive analysis of self-efficacy

It can be seen from the data shown in Table 5 that the average value of self-efficacy is 28.062, while the median is 32.000, and the average value is significantly lower than the median. At the same time, the standard deviation of junior high school students' self-efficacy is 7.401, which is relatively large. Combined with the range from the minimum value of 11 to the maximum value of 38, it indicates that there are significant individual differences among junior high school students in terms of self-efficacy.

**Table 5:** Descriptive statistics of self-efficacy among junior secondary school students (*n* = 729).

Variable	Min	Max	Mean	SD	Median
Self-efficacy	11.000	38.000	28.062	7.401	32.000

#### 4.2.4 Descriptive analysis of physical exercise behavior

It can be seen from the data shown in Table 6 that the mean score of physical exercise behavior was 33.653, with a median of 30.000. The minimum value was 0.000, and the SD reached 26.198.

**Table 6:** Descriptive statistics of physical activity behaviors among junior high school students (*n* = 729).

Variable	Min	Max	Mean	SD	Median
Physical exercise behavior	0.000	100.000	33.653	26.198	30.000

### 4.3 The effect of differences in demographic variables on the family sports environment, perceived family support, self-efficacy and physical exercise behavior of junior high school students

It can be seen from the tabular data that there is no significant difference in the family physical environment of junior high school students with different family structures (*p* > 0.05). There are significant differences in the family physical behavior environment, family physical psychological environment, and family physical environment total scores of junior high school students with different family structures (*p* < 0.05). In general, the gender, grade, number of children in the family and family structure of junior high school students will affect the creation of a family physical environment (Tables 7–10).

**Table 7:** Statistics of differences in family sports environment among junior high school students by gender.

Categories	Boys (*n* = 372)	Girls (*n* = 357)	*t*	*p*
Family physical behavior environment	131.35 ± 27.11	127.43 ± 28.96	1.886	0.060
Family physical education environment	93.83 ± 20.18	90.28 ± 21.17	2.316	0.021^**^
Family physical psychology environment	55.10 ± 12.62	52.89 ± 13.32	2.291	0.022^**^
Family physical environment	280.28 ± 58.62	270.60 ± 62.38	2.156	0.031^**^

^*^p < 0.05, ^**^p < 0.01.

**Table 8:** Statistics of differences in family sports environment among junior middle school students by grade.

Categories	Grade 1 (*n* = 259)	Grade 2 (*n* = 224)	Grade 3 (*n* = 246)	*F*	*p*	LSD
Family physical behavior environment	131.84 ± 27.06	127.40 ± 28.81	128.74 ± 28.38	1.617	0.199	–
Family physical education environment	93.65 ± 20.12	90.07 ± 21.03	92.30 ± 21.02	1.819	0.163	–
Family physical psychology environment	55.19 ± 12.80	52.25 ± 12.92	54.39 ± 13.17	3.236	0.040^**^	Grade1 > Grade2
Family physical environment	280.68 ± 58.74	269.72 ± 61.74	275.42 ± 61.36	1.969	0.140	–

^*^p < 0.05, ^**^p < 0.01.

**Table 9:** Statistics of differences in family sports environment among junior middle school students with different number of children.

Categories	The only child (*n* = 375)	Non-only child (*n* = 354)	*t*	*p*
Family physical behavior environment	131.02 ± 27.80	127.74 ± 28.31	1.580	0.115
Family physical education environment	93.12 ± 20.46	91.01 ± 20.99	1.374	0.170
Family physical psychology environment	55.05 ± 12.85	52.93 ± 13.10	2.202	0.028^**^
Family physical environment	279.19 ± 59.88	271.68 ± 61.29	1.672	0.095

^*^p < 0.05, ^**^p < 0.01.

**Table 10:** Statistics of differences in family sports environment among junior middle school students with different family structures.

Categories	Nuclear family (*n* = 196)	Stem family (*n* = 298)	Single parent family (*n* = 133)	Intergenerational family (*n* = 102)	*F*	*p*	LSD
Family physical behavior environment	132.02 ± 26.06	127.33 ± 29.65	125.70 ± 28.98	135.43 ± 24.64	3.484	0.016^**^	Nuclear family > Single parent family Intergenerational family > Stem family Intergenerational family > Single parent family
Family physical education environment	93.58 ± 19.95	91.06 ± 21.29	89.47 ± 21.76	95.69 ± 18.65	2.325	0.074	–
Family physical psychology environment	55.79 ± 11.27	52.76 ± 13.90	51.96 ± 13.59	56.98 ± 11.79	5.096	0.002^**^	Nuclear family > Stem family Nuclear family > Single parent family Intergenerational family > Stem family Intergenerational family > Single parent family
Family physical environment	281.38 ± 56.13	271.15 ± 63.69	267.13 ± 62.91	288.10 ± 54.29	3.473	0.016^**^	Nuclear family > Single parent family Intergenerational family > Stem family Intergenerational family > Single parent family

^*^p < 0.05, ^**^p < 0.01.

It can be seen from the tabular data that there are significant differences in the perceived family support of junior high school students with different family structures (*p* < 0.05). In general, there are significant differences among students of different genders and family structures in perceived family support (Tables 11–14).

**Table 11:** Statistics on the differences in perceived family support between male and female junior middle school students.

Categories	Boys (*n* = 372)	Girls (*n* = 357)	*t*	*p*
Perceived family support	18.98 ± 5.80	18.00 ± 6.22	2.182	0.029^**^

^*^p < 0.05, ^**^p < 0.01.

**Table 12:** Statistics of differences in perceived family support among junior middle school students by grade.

Categories	Grade 1 (*n* = 259)	Grade 2 (*n* = 224)	Grade 3 (*n* = 246)	*F*	*p*	LSD
Perceived family support	18.91 ± 5.83	18.42 ± 6.09	18.14 ± 6.18	1.045	0.352	–

^*^p < 0.05, ^**^p < 0.01.

**Table 13:** Statistics on differences in perceived family support among junior middle school students with different number of children.

Categories	The only child (*n* = 375)	Non-only child (*n* = 354)	*t*	*p*
Perceived family support	18.69 ± 6.09	18.30 ± 5.96	0.870	0.385

^*^p < 0.05, ^**^p < 0.01.

**Table 14:** Statistics on differences in perceived family support among junior middle school students by family structure.

Categories	Nuclear family (*n* = 196)	Stem family (*n* = 298)	Single parent family (*n* = 133)	Intergenerational family (*n* = 102)	*F*	*p*	LSD
Perceived family support	19.06 ± 5.89	18.03 ± 6.31	17.47 ± 5.87	20.12 ± 5.22	4.977	0.002^**^	Nuclear family > Single parent family Intergenerational family > Stem family Intergenerational family > Single parent family

^*^p < 0.05, ^**^p < 0.01.

It can be seen from the tabular data, there are significant differences in the self-efficacy of junior high school students with different family structures (*p* < 0.05). In general, there are significant differences in self-efficacy among students of different genders and family structuresc (Tables 15–18).

**Table 15:** Statistical differences of self-efficacy between male and female junior high school students.

Categories	Boys (*n* = 372)	Girls (*n* = 357)	*t*	*p*
Self-efficacy	28.61 ± 7.20	27.49 ± 7.57	2.035	0.042^**^

^*^p < 0.05, ^**^p < 0.01.

**Table 16:** Statistical differences of self-efficacy among junior middle school students by grade.

Categories	Grade 1 (*n* = 259)	Grade 2 (*n* = 224)	Grade 3 (*n* = 246)	*F*	*p*	LSD
Self-efficacy	28.79 ± 7.15	27.36 ± 7.51	27.93 ± 7.52	2.319	0.099	–

^*^p < 0.05, ^**^p < 0.01.

**Table 17:** Statistical differences of self-efficacy among junior middle school students with different number of children.

Categories	The only child (*n* = 375)	Non-only child (*n* = 354)	*t*	*p*
Self-efficacy	28.43 ± 7.53	27.67 ± 7.25	1.401	0.162

^*^p < 0.05, ^**^p < 0.01.

**Table 18:** Statistics on differences in self-efficacy among junior middle school students by family structure.

Categories	Nuclear family (*n* = 196)	Stem family(*n* = 298)	Single parent family (*n* = 133)	Intergenerational family (*n* = 102)	*F*	*p*	LSD
Self-efficacy	28.97 ± 7.13	27.35 ± 7.68	27.08 ± 7.39	29.69 ± 6.67	4.390	0.005^**^	Nuclear family > Stem family Nuclear family > Single parent family Intergenerational family > Stem family Intergenerational family > Single parent family

^*^p < 0.05, ^**^p < 0.01.

It can be seen from the tabular data in Tables 19–22, there are significant differences in the physical exercise behavior of junior high school students with different family structures (*p* < 0.05). In general, there are significant differences in physical exercise behavior among students with different family structures and junior high school students from nuclear families have better physical exercise behavior than junior high school students from other structural families.

**Table 19:** Statistical differences of physical exercise behaviors between male and female junior middle school students.

Categories	Boys (*n* = 372)	Girls (*n* = 357)	*t*	*p*
Physical exercise behavior	34.35 ± 27.00	32.93 ± 25.35	0.730	0.466

^*^p < 0.05, ^**^p < 0.01.

**Table 20:** Statistical differences of physical exercise behaviors among junior middle school students in different grades.

Categories	Grade 1 (*n* = 259)	Grade 2 (*n* = 224)	Grade 3 (*n* = 246)	*F*	*p*	LSD
Physical exercise behavior	34.80 ± 25.59	31.13 ± 24.32	34.75 ± 28.34	1.508	0.222	–

^*^p < 0.05, ^**^p < 0.01.

**Table 21:** Statistical differences in physical exercise behavior among junior middle school students with different number of children.

Categories	The only child (*n* = 375)	Non-only child (*n* = 354)	*t*	*p*
Physical exercise behavior	35.29 ± 27.08	31.92 ± 25.15	1.734	0.083

^*^p < 0.05, ^**^p < 0.01.

**Table 22:** Statistics of differences in physical exercise behaviors among junior middle school students with different family structures.

Categories	Nuclear family (*n* = 196)	Stem family (*n* = 298)	Single parent family (*n* = 133)	Intergenerational family (*n* = 102)	*F*	*p*	LSD
Physical exercise behavior	39.29 ± 29.29	31.20 ± 24.07	31.79 ± 28.60	32.41 ± 20.81	4.253	0.005^**^	Nuclear family > Stem family Nuclear family > Single parent family Nuclear family > Intergenerational family

^*^p < 0.05, ^**^p < 0.01.

### 4.4 Correlation analysis of family sports environment, perceived family support, self-efficacy and physical exercise behavior

Before Pearson correlation analysis, collinearity diagnostics were performed for family sports environment (and its sub-dimensions), perceived family support, self-efficacy, and physical exercise behavior. A VIF value greater than 10 indicates severe collinearity. As shown in Table 23, all VIF values were below 10 and all tolerance values were above 0.1, indicating no serious multicollinearity among variables.

**Table 23:** Collinearity diagnostics.

Categories	VIF	Tolerance
Self-efficacy	1.862	0.537
Perceived family support	1.551	0.645
Family sports behavioral environment	1.840	0.543
Family sports physical environment	1.797	0.557
Family sports psychological environment	1.804	0.554
Family sports environment (total)	1.878	0.533

It can be seen from the data shown in Table 24 that all correlation coefficients were above 0.59 and significant at *p* < 0.01, indicating significant positive correlations. Physical exercise behavior was positively predicted by family sports environment, perceived family support, and self-efficacy, with the overall family sports environment being especially influential.

**Table 24:** Pearson correlation between variables and physical exercise behavior.

Categories	Physical exercise behavior
Self-efficacy	0.680
Perceived family support	0.596
Family sports behavioral environment	0.676
Family sports physical environment	0.666
Family sports psychological environment	0.668
Family sports environment (total)	0.684

It can be seen from the data shown in Table 25 that all pairwise correlations were significant at *p* < 0.01, showing strong positive relationships. Family sports environment, perceived family support, and self-efficacy were mutually reinforcing. As a foundational external factor, family sports environment shaped both perceived support and self-efficacy, which in turn promoted internal motivation for physical exercise.

**Table 25:** Pearson correlation analysis of family sports environment, perceived family support and self-efficacy.

Categories	Family sports behavior environment	The physical environment of family sports	Family sports psychological environment	Family sports environment	Perceived family support	self-efficacy
Family sports behavioral environment	1					
Family sports physical environment	0.950	1				
Family sports psychological environment	0.934	0.931	1			
Family sports environment (total)	0.988	0.981	0.965	1		
Perceived family support	0.870	0.875	0.851	0.885	1	
Self-efficacy	0.940	0.937	0.917	0.952	0.845	1

### 4.5 Effects of family sports environment, perceived family support and self-efficacy on physical exercise behavior

AMOS 24.0 was used to construct structural equation models with family sports environment, perceived family support, and self-efficacy as independent variables, and physical exercise behavior as the dependent variable. Model fit and path coefficients were examined.

It can be seen from the data shown in Table 26 that all fitting indicators meet the requirements of ideal values or standard values, indicating that the constructed structural equation model of “the impact of family sports environment on physical exercise behavior” has a good overall fit, the model setting is reasonable, and it can better reflect the actual relationship between variables.

**Table 26:** Path-model fit indicators for analyzing the impact of family sports environment on physical exercise behavior.

Index	CMIN	DF	CMIN/DF	GFI	RMSEA	CFI	NFI	IFI
Ideal	–	–	< 3	>0.9	< 0.08	>0.9	>0.9	>0.9
Acceptable	–	–	< 5	>0.8	< 0.10	>0.8	>0.8	>0.8
Estimate	650.997	149	4.369	0.919	0.068	0.964	0.954	0.964

From the data shown in Table 27, it can be seen that the unstandardized path coefficient is 0.776, the standardized path coefficient is 0.691, the C.R. (critical ratio) is 22.896, and the *p*-value is less than 0.01, reaching an extremely significant level. This indicates that the family sports environment has a significant and strong positive impact on sports exercise behavior. For each unit increase in the family sports environment, the sports exercise behavior increases by an average of 0.776 units. Families can directly and effectively improve junior high school students' sports exercise behavior by creating a good sports atmosphere, providing sports equipment and space, and parents setting an example by participating in exercise.

**Table 27:** Summary of model coefficients of the influence analysis of family sports environment on physical exercise behavior.

Independent variable	Dependent variable	Unstandardized path coefficient	Standardized path coefficient	Standard error	C.R.	*P*
Family sports environment	Physical exercise behavior	0.776	0.691	0.034	22.896	0.000^**^

^*^p < 0.05, ^**^p < 0.01.

It can be seen from the data shown in Tables 28 and 29 that perceived family support significantly and positively predicted physical exercise behavior (β = 0.622, *p* < 0.01). A one-unit increase in perceived support predicted a 0.695 increase in exercise behavior. Subjective perceived support was critical for sustained exercise.

**Table 28:** Path analysis of the impact of perceived family support on physical exercise behavior-model fit index.

Ideal value	–	–	< 3	>0.9	< 0.08	>0.9	>0.9	>0.9
Standard-reaching value	–	–	< 5	>0.8	< 0.10	>0.8	>0.8	>0.8
Fitted value	23.765	5	4.753	0.987	0.072	0.968	0.961	0.969

**Table 29:** Summary of model coefficients for the analysis of the impact of perceived family support on physical exercise behavior.

Independent variable	Dependent variable	Unstandardized path coefficient	Standardized path coefficient	Standard error	C.R.	*P*
Perceive family support	Physical exercise behavior	0.695	0.622	0.058	11.971	0.000^**^

^*^p < 0.05, ^**^p < 0.01.

It can be seen from the data shown in Table 30 and 31 that self-efficacy had the strongest positive effect (β = 0.708, *p* < 0.01). A one-unit increase in self-efficacy predicted a 1.030 increase in exercise behavior. This supported Bandura's social cognitive theory: self-efficacy is a key determinant of behavior initiation and persistence.

**Table 30:** Path analysis of the influence of self-efficacy on physical exercise behavior-Model fit index.

Indicator	CMIN	DF	CMIN/DF	GFI	RMSEA	CFI	NFI	IFI
Ideal value	–	–	< 3	>0.9	< 0.08	>0.9	>0.9	>0.9
Compliance value	–	–	< 5	>0.8	< 0.10	>0.8	>0.8	>0.8
Fitted value	161.642	44	3.674	0.961	0.061	0.975	0.966	0.975

**Table 31:** Summary of model coefficients for the analysis of the influence of self-efficacy on physical exercise behavior.

Independent variable	Dependent variable	Unstandardized path coefficient	Standardized path coefficient	Standard error	C.R.	*P*
Perceive family support	Physical exercise behavior	1.030	0.708	0.053	19.286	0.000^**^

^*^p < 0.05, ^**^p < 0.01.

### 4.6 Testing the mediating effect of junior high school students' family sports environment, perceived family support, self-efficacy, and physical exercise behavior

In the overall sample, perceived family support and self-efficacy showed no significant mediating or chain mediating effects. Mediation paths were only significant in stem families: (1) negative indirect effect: family sports environment → perceived family support → physical exercise behavior; (2) positive indirect effect: family sports environment → self-efficacy → physical exercise behavior. The full chain mediation path was not significant in the total sample or any family subgroup. Only two separate mediation paths were significant in stem families. All these are shown in Table 32.

**Table 32:** Results of the mediation effect test for stem families.

Item	Symbol	Meaning	Effect size effect	95% CI		Conclusion
				Lower limit	Upper limit	
Family sports environment → Perceived family support → Sports exercise behavior	a^*^b	Indirect effect	−1.786	−1.991	−1.567	Some intermediaries
Family sports environment → Perceived family support	a	X = >M	1.739	1.689	1.789	
Perceived family support → Sports exercise behavior	b	M = >Y	−1.027	−1.179	−0.875	
Family sports environment → sports exercise behavior	c'	direct effect	1.050	0.898	1.202	
Family sports environment → sports exercise behavior	c	Total effect	2.836	−1.093	−0.365	

## 5 Discussion

### 5.1 Characteristics of junior high school students' family sports environment, perceived family support, self-efficacy, and physical exercise behavior

#### 5.1.1 Characteristics of the family sports environment

This study found that the overall level of the family sports environment of junior high school students is at a moderate to low level, with the mean score lower than the median, and there are obvious individual differences among students. Among the three dimensions, the psychological environment has the lowest score, followed by the physical environment, which is consistent with the research conclusions of Zheng (2019) and Zhang (2024). From the perspective of social cognitive theory, the family sports environment, as an important external situational factor, its imperfection will directly affect the formation of adolescents‘ sports cognition and exercise behavior. In terms of the behavioral environment, the phenomenon of parents holding the “score-first” concept is still prominent. Some parents have realized the importance of the family sports environment, but on the whole, there are still problems of insufficient behavioral, physical, and psychological environments, among which the lack of physical and psychological environments is more prominent. In terms of the behavioral environment, some parents set an example to encourage their children to exercise, but there are still parents who restrict their children's sports activities due to the influence of the “only- score theory”; in the physical environment, most families have basic equipment such as skipping ropes, but the allocation rate of professional equipment is low, and community public sports facilities have problems such as being outdated and insufficient in quantity. Some families have idle equipment due to insufficient space, economy, or guidance; in the psychological environment, parents' cognition of sports mostly stays at physical fitness.

#### 5.1.2 The characteristics of perceived family support

The overall level of perceived family support among junior high school students is acceptable but not high. Some students have long been in a state of support deprivation, which affects their mental health and sports participation. This research result is consistent with the views of researchers such as Liu (2023) and Yang (2024). The current family support has a “grades first” orientation. Parents focus more on academic support, pay insufficient attention to non-academic fields such as sports, and even restrict their children's physical exercise, leading to a decrease in students' perceived support. Junior high school students desire equal communication and respect, but some families still use an authoritative parenting model, lacking effective communication, resulting in a parent-child generation gap. There are individual differences in perceived family support: boys perceive a higher degree of support than girls; students from intergenerational families perceive stronger support because their grandparents focus more on emotional care and impose less academic pressure. The effectiveness of support lies in the child's “perception,” and the core is the quality of emotional connection.

#### 5.1.3 Characteristics of self-efficacy

Junior high school students have a certain level of self-efficacy, but there are significant individual differences, showing a trend of “polarization”: some students have strong confidence in sports, can actively set goals and meet challenges; others lack confidence, have a fear of difficulty in physical education, and are afraid of failure. In terms of gender, boys' self-efficacy is significantly higher than that of girls, which is highly consistent with the research results of scholars such as Mao (2003), and Chen and Zhou (2007). This is because boys participate in physical exercise more frequently, especially prefer competitive sports, and have accumulated more successful experiences. Moreover, their physiological advantages after puberty make them easily recognized; girls participate in physical exercise with lower frequency and intensity, avoid strenuous exercise due to concerns about their image, etc., resulting in insufficient successful experiences. In terms of family structure, students from intergenerational families have higher self-efficacy. The emotional tolerance, support from grandparents, and the teaching of traditional sports games help children accumulate successful experiences and enhance their confidence.

#### 5.1.4 The characteristics of physical exercise behavior

The overall physical exercise behavior of junior high school students is not ideal, and there is a gap from the ideal state of health promotion. This research result is consistent with the views of researchers such as Zheng (2019) and Zhang (2024). According to the scale classification, 39.51% of the students meet the standard of large amount of exercise, 22.63% have a moderate amount of exercise, and 37.86% have a small amount of exercise. More than 60% of the students are at the level of moderate or small amount of exercise. Physical exercise behavior is closely related to family background. Students from nuclear families have relatively better exercise behavior because their family functions are stable, communication is efficient, and parents have more advantages in resource input, activity planning and supervision. However, single-parent families, intergenerational families, etc., have weak relevant support due to limitations in resources, physical strength or concepts. It is necessary to focus on the group with small amount of exercise, especially students from families with weak support, and formulate precise intervention strategies.

### 5.2 The relationship between junior high school students' family sports environment, perceived family support, self-efficacy, and physical exercise behavior

#### 5.2.1 Correlation and predictive effects

According to relevant analyses, it has been found that the physical exercise behavior of junior high school students is significantly and positively influenced by the family sports environment, perceived family support, and self-efficacy. Structural equation modeling further verified the positive linear relationships between the family sports environment, perceived family support, self-efficacy and physical exercise behavior, respectively. This research conclusion is consistent with the viewpoints of Zheng (2019), Zhang et al. (2018), and Yao (2015) and others. The study shows that the physical exercise behavior of junior high school students is significantly and positively affected by the family sports environment, perceived family support, and self-efficacy, and there are significant positive correlations between each pair of the three, forming an interactive mechanism of mutual promotion. Among them, the role of the family sports environment is particularly prominent. It is not only reflected in parents' behavioral demonstrations and material guarantees, but also includes recognition of the value of sports and spiritual support. Students with higher scores in this aspect also have higher frequency, intensity, and duration of physical exercise. Perceived family support can provide students with emotional security and confidence in coping, making it easier for them to persist in exercise when facing obstacles. As an internal driving force, self-efficacy can enhance students' initiative and persistence in exercise, prompting them to try new sports and set high goals. At the same time, a good family sports environment can enhance students' perception of family support, a high level of perceived family support can improve self-efficacy, and students with high self-efficacy will also actively optimize the interaction quality of the family sports environment. This conclusion is consistent with the viewpoints of many researchers.

#### 5.2.2 Mediating and chain mediating effects

The most important finding of this study is that the mediating effect and chain mediating effect of perceived family support and self-efficacy are group heterogeneous, which is the key supplement to the existing research. Studies have found that at the overall sample level, there is no significant mediating effect among family sports environment, perceived family support, and physical exercise behavior. However, when further analyzing by family structure, it was found that only in stem families, there exists the mediating path of “family sports environment → perceived family support → physical exercise behavior”, and it is a significant negative indirect effect. The study also found that in the overall sample, there is no significant mediating relationship among family sports environment, self-efficacy, and physical exercise behavior. The chain mediation model was not supported in the full sample. Mediation effects were only present in stem families, with family structure acting as a critical boundary condition. According to social cognitive theory, the environment is an important external variable affecting individual behavior. It not only provides the physical conditions for behavior to occur, but also influences individual cognition and motivation through information.

### 5.3 Theoretical implications

This study has important theoretical contributions to the research field of adolescent physical exercise behavior in sport psychology: first, based on social cognitive theory, this study constructs and verifies the family sports environment-perceived family support-self-efficacy-physical exercise behavior chain mediation model. Second, this study reveals the group heterogeneity of the mediating effect of perceived family support and self-efficacy, and confirms that family structure is an important boundary condition for the transmission of the environment-perception-motivation-behavior chain. This finding breaks the limitation of existing research that ignores the group difference of the mediating effect, and provides a new research perspective for the subsequent study on the influence mechanism of adolescent physical exercise behavior. Third, this study further clarifies the hierarchical relationship among family sports environment, perceived family support and self-efficacy in the influence of physical exercise behavior: the family sports environment is the foundational external variable, perceived family support is the intermediate psychological perception variable, and self-efficacy is the core internal motivation variable. This hierarchical relationship makes the theoretical framework of the influence of family factors on adolescent physical exercise behavior more clear and systematic.

## 6 Conclusion

The family sports environment, perceived family support, and self-efficacy of junior high school students are all at a relatively low level. Their sports exercise behavior is poor. There are significant differences in these variables among different demographic variables. All three variables positively influence the sports exercise behavior of junior high school students. Among them, the family sports environment has the strongest effect, and all three variables are significantly positively correlated with each other. The mediating and chain mediating paths only exist in stem families, not in the full sample or other family structure groups. It is suggested to popularize the value of sports education through parent schools, guide parents to create a good family sports environment, set short-term exercise goals for children, pay more attention to progress and give sincere praise, and enhance their perception of family support and self-efficacy. Schools should provide differentiated sports guidance for different family structures such as core, main, single-parent, and step-parent families. Communities should open public sports facilities and hold family sports activities to jointly build a sports support network of family, school, and community.

## Data Availability

The original contributions presented in the study are included in the article/supplementary material, further inquiries can be directed to the corresponding author/s.
